# Navigating the Esophagus: Effective Strategies for Foreign Body Removal

**DOI:** 10.7759/cureus.38593

**Published:** 2023-05-05

**Authors:** Rajmohan Rammohan, Melvin Joy, Dilman Natt, Tulika Saggar, Sai Greeshma Magam, Sandra Gomez, Najia Sayedy, Jiten Desai, Susan Bunting, Paul Mustacchia

**Affiliations:** 1 Gastroenterology, Nassau University Medical Center, East Meadow, USA; 2 Internal Medicine, Nassau University Medical Center, East Meadow, USA; 3 Gastroenterology and Hepatology, Nassau University Medical Center, East Meadow, USA; 4 Pulmonary and Critical Care, Nassau University Medical Center, East Meadow, USA

**Keywords:** ent procedures, geriatric psychiatry, urgent upper endoscopy, emergency gastroenterology and endoscopy, esophageal foreign body

## Abstract

Foreign body ingestion is a common medical emergency that can affect individuals of all ages and can be caused by various factors, including accidental ingestion, psychiatric disorders, intellectual disabilities, and substance abuse. The most common site for foreign body lodgment is the upper esophagus, followed by the middle esophagus, stomach, pharynx, lower esophagus, and duodenum. This article provides a case report of a 43-year-old male patient with a history of schizoaffective disorder and an indwelling suprapubic catheter who presented to the hospital due to foreign body ingestion. After examination, a metal clip from his Foley catheter was found lodged in his esophagus. The patient was intubated for the procedure, and an emergent endoscopic removal was performed to remove the metallic Foley component. No postoperative complications were observed, and the patient was successfully discharged.

This case highlights the importance of considering foreign body ingestion in patients with chest pain, dysphagia, and vomiting. Prompt diagnosis and treatment are crucial to prevent potential complications such as perforation or gastrointestinal tract obstruction. The article also emphasizes the need for healthcare providers to know the different risk factors, variations, and common sites for foreign body lodgment to optimize patient care. Furthermore, the article highlights the importance of multidisciplinary care involving psychiatry and surgery to provide comprehensive care to patients with psychiatric disorders who may be at higher risk for foreign body ingestion. In conclusion, foreign body ingestion is a typical medical emergency that requires prompt diagnosis and treatment to prevent complications. This case report highlights the successful management of a patient with foreign body ingestion and emphasizes the importance of multidisciplinary care to optimize patient outcomes.

## Introduction

Foreign bodies refer to any object that comes from outside an organism's body, and in machinery, it denotes an undesirable object that interferes with normal functioning. Among humans, foreign bodies are commonly found in the alimentary tract, making it one of the most common locations [1]. Therefore, ingesting foreign bodies is a joint presentation in emergency departments worldwide, affecting individuals of all ages.

Adults with psychiatric disorders, intellectual disabilities, suicidal tendencies, or acute intoxication from alcohol or illicit drug abuse, as well as prisoners or body packers (people who transport illegal drugs by ingesting them), are more vulnerable [2-4]. Meanwhile, curiosity or accidental ingestion is the most common cause in children, with a peak incidence between six months to three years old [5]. Additionally, foreign body ingestion is more prevalent in males, with some studies indicating a male-to-female ratio of about 1.5 to 1 [6-7].

Foreign body ingestion can be categorized into two primary types: true foreign object ingestion (FOI) and esophageal food impaction (EFI) [8]. The geographic variation in FOI epidemiology is remarkable, with different substances consumed and patient demographic variations [9]. For example, Americans commonly experience food (meat) impaction [9-10], whereas, in other countries, it's fishbone [11-17]. 

The upper esophagus is the most common site for foreign body lodgment, followed by the middle esophagus, stomach, pharynx, lower esophagus, and finally, the duodenum [8]. Foreign body ingestion is a common occurrence that can occur in anyone, with different risk factors and variations depending on the patient's age, gender, and geographic location. Knowing the common sites for foreign body lodgment can help physicians diagnose and manage.

## Case presentation

**Figure 1:**
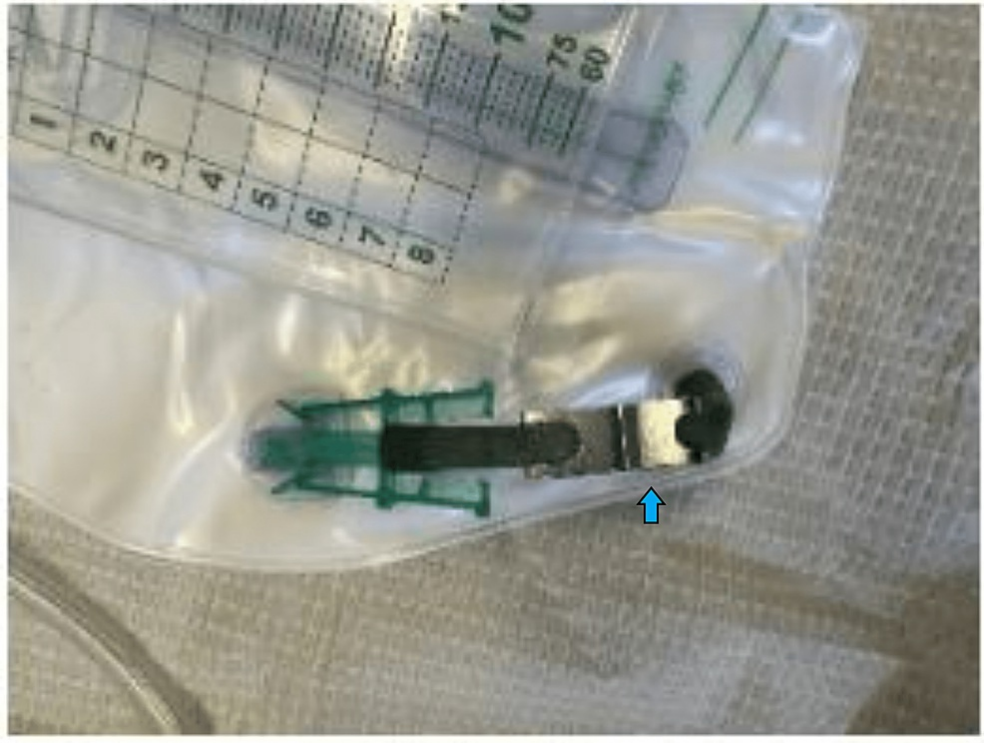
Metallic clip from the Foley bag

**Figure 2:**
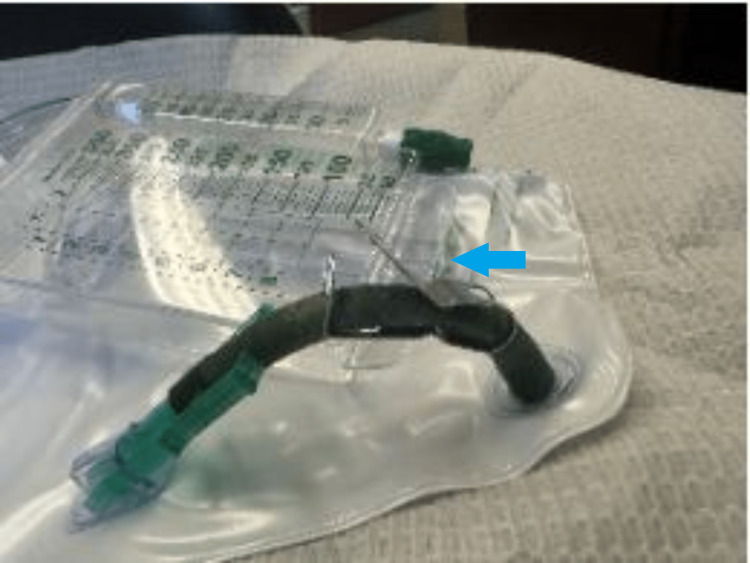
Metallic clip from the Foley bag lateral view

**Figure 3:**
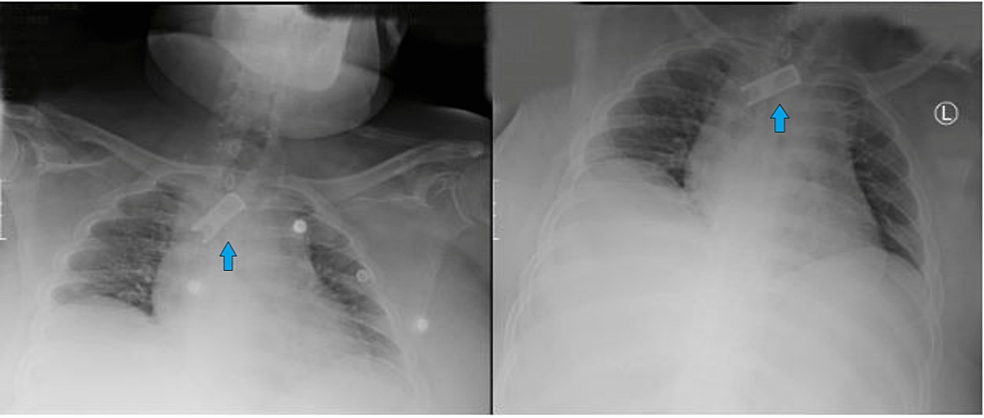
Pre-endoscopy X-ray image showing the foreign body

A 43-year-old male patient with a past psychiatric history of schizoaffective disorder and a lifelong indwelling suprapubic catheter due to chronic retention presented to the hospital due to foreign body ingestion (Figures 1, 2). After examination, a metal clip from his Foley catheter was lodged in his esophagus. Despite being bed bound since childhood, the patient was vitally stable and maintained his airway upon presentation. A chest x-ray revealed a rectangular metallic object measuring 4.7cm x 1.8cm projecting over the central superior mediastinum (Figure 3). No evidence of subcutaneous emphysema was noted on the radiograph. 

**Figure 4:**
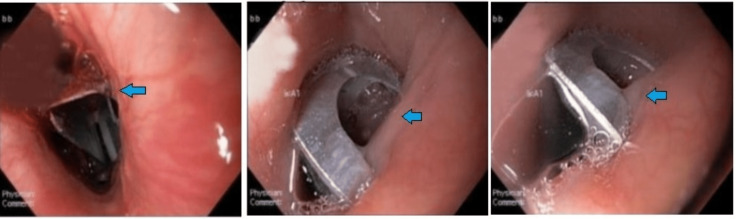
Upper endoscopy showing foreign body in the upper esophagus

**Figure 5:**
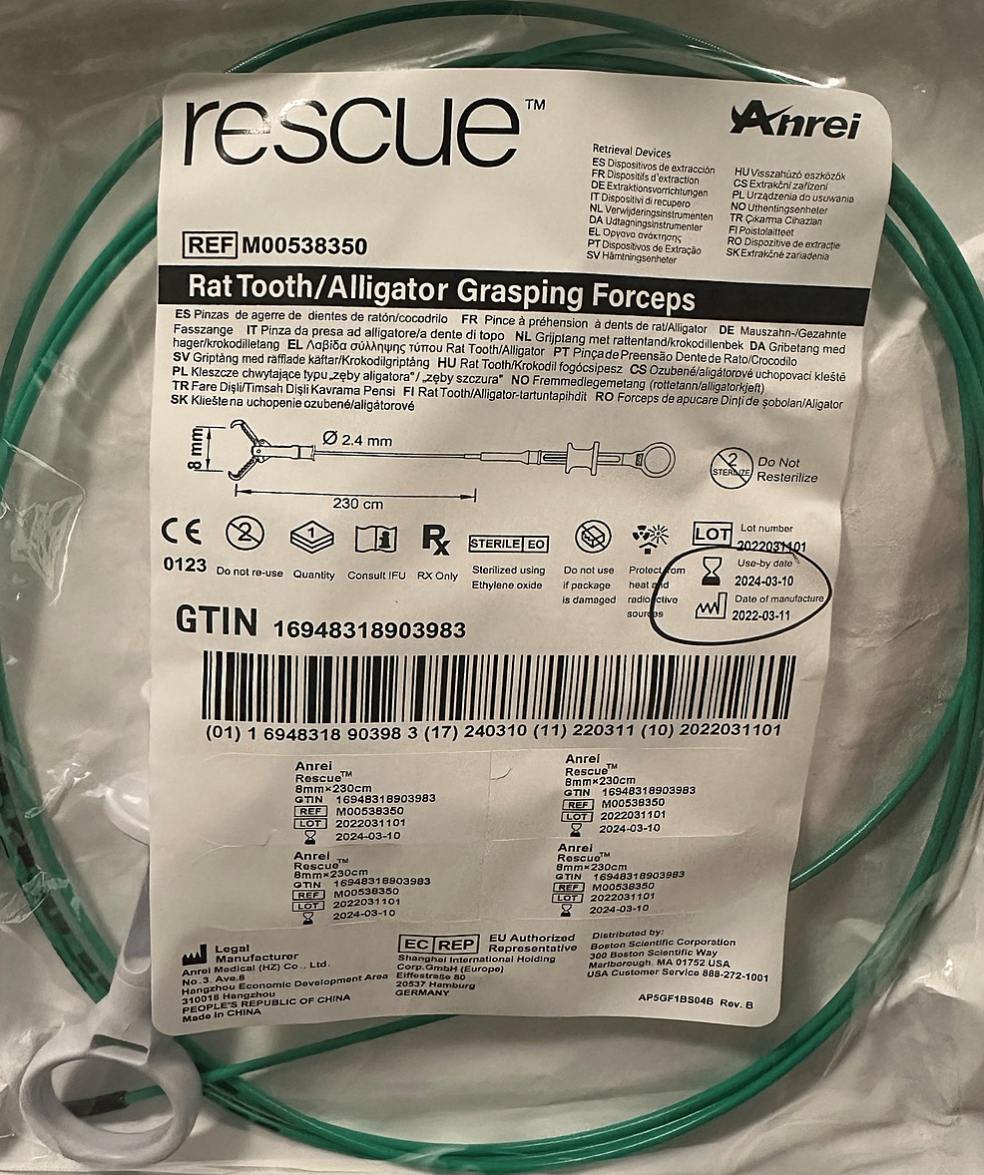
Rat tooth/alligator forceps used for the removal of the foreign body

The medical team planned an emergent endoscopic removal of the metallic Foley component and intubated the patient for the procedure. A foreign body was noted during the upper endoscope at 25cm from the incisors (Figure 4). Using 8mm rat tooth forceps (Anrei, Hangzhou, China) (Figure 5), the edges of the metallic object were grasped and pulled gently in a corkscrew fashion, with no resistance, until it reached the level of the cricopharyngeal muscle. Resistance was met at this point, and subsequent laryngoscopy was performed for further removal.

**Figure 6:**
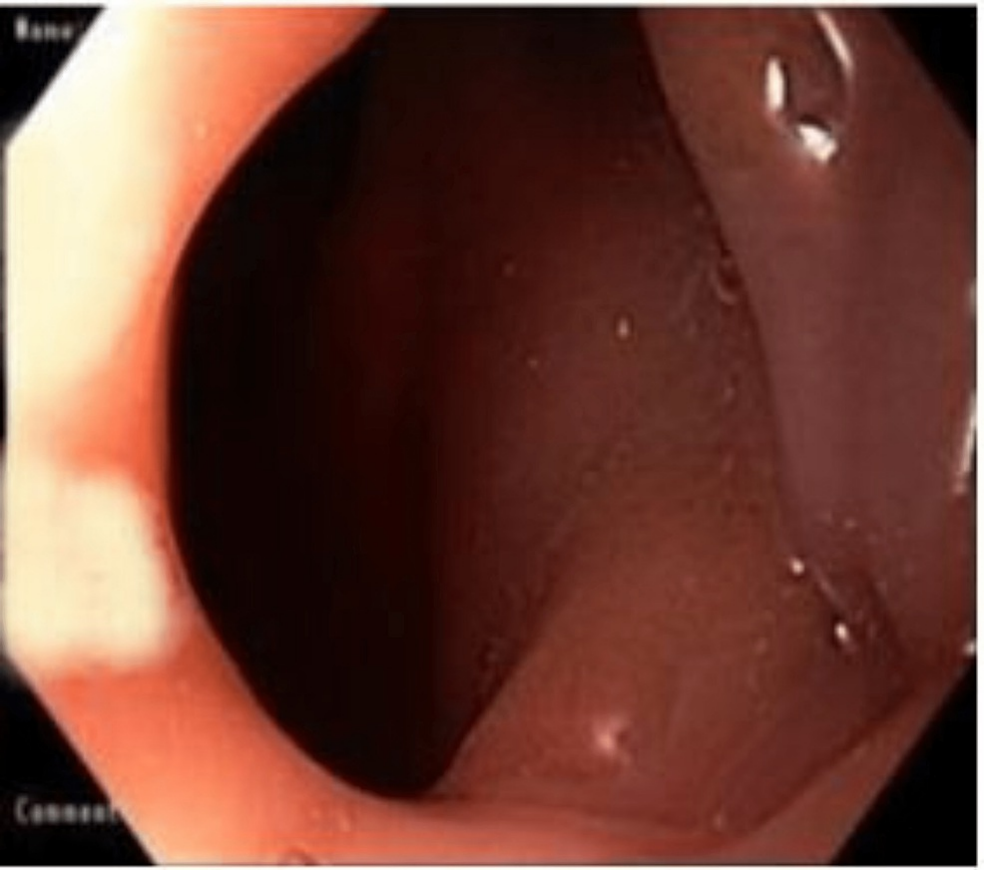
Upper endoscopy image post foreign body removal

**Figure 7:**
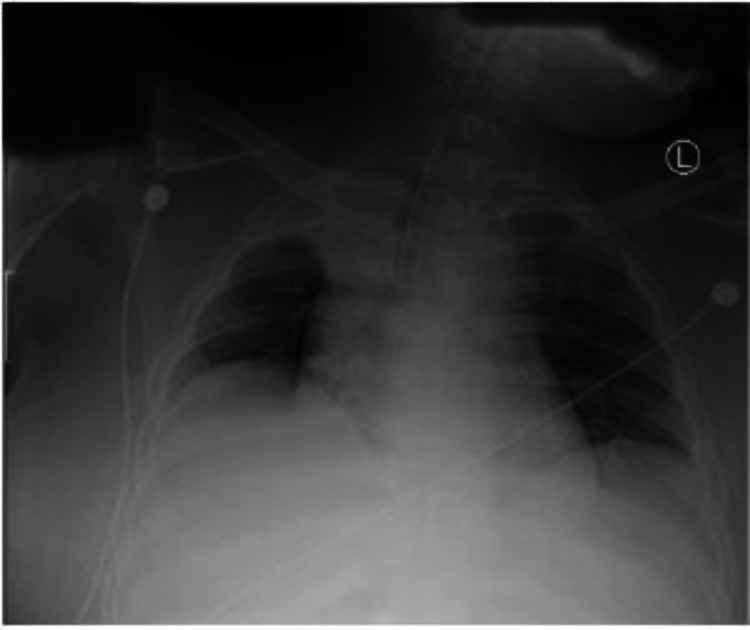
X-ray image - post foreign body removal

After successfully retrieving the foreign body, the mucosa was examined, and a small amount of bleeding was noted, but no gross lacerations or perforations were seen. As a precautionary measure, approximately 8cc of 1:100,000 epinephrine was sprayed in the area, and lidocaine with epinephrine was sprayed at the site of the foreign body removal to minimize bleeding secondary to mucosal irritation. Figure 6 shows the post-removal endoscopic view. A postoperative chest x-ray was performed to rule out pneumomediastinum and pneumothorax, as shown in Figure 7. The patient was then extubated successfully, and no postoperative complications were observed. Empiric antibiotics were administered, and the patient remained clinically and hemodynamically stable.

Speech and swallow evaluation was conducted, and oral feeds were initiated as the patient tolerated. Psychiatry was consulted and provided recommendations regarding optimizing the patient's psychiatric medications. The patient's home medications of olanzapine 20mg, Seroquel 300mg, and trazodone 50mg were resumed. The patient was followed by the Medical Intensive Care Unit, Surgery (ENT), during admission and cleared for discharge.

## Discussion

Diagnosis of foreign body ingestion

Foreign body ingestion and impaction can cause significant morbidity and even mortality. Therefore, a thorough history and physical examination are crucial in diagnosing such cases. The history should include the time of foreign body ingestion, the type of foreign body, the onset of symptoms, the location of discomfort, and the severity of symptoms such as dysphagia, drooling, choking, stridor, wheezing, dysphonia, odynophagia, and respiratory distress. These severe signs may indicate airway obstruction or esophageal perforation, which require immediate intervention. However, psychiatrically impaired individuals and younger children make it difficult to take a good history, and a simple radiograph can provide insight and aid in the diagnosis [18].

Physical examination should include an inspection of the oro- and hypopharynx, neck, chest, and abdomen to detect esophageal obstruction or perforation, which may present as cervical swelling/crepitus in case of oropharyngeal/proximal esophageal perforation or fever/peritonitis in case of intestinal perforation [8,19]. For initial diagnosis in non-emergent situations, plain radiographs can confirm the location, size, shape, and number of radiopaque foreign bodies ingested and exclude aspirated objects. However, radiographs cannot detect radiolucent objects. Metal detectors can detect most metal objects ingested, including radiolucent metallic foreign bodies like aluminum, and benefit pediatric patients [20-24].

Computed tomography (CT) scanning can identify an impacted foreign body's precise location, shape, size, and depth and help visualize its surrounding tissue. CT scan provides better anatomical details and can detect other complications such as abscess/tracheal fistula and mediastinitis. Three-dimensional reconstruction can enhance the sensitivity and accuracy of CT scans [25]. However, contrast administration should typically not be attempted due to the risk of contrast aspiration and the potential for compromising any subsequent endoscopy [26]. Prompt and appropriate management can prevent complications and ensure better patient outcomes.

Endoscopy retrieval instruments

In cases where foreign bodies are ingested or lodged in the airway, prompt evaluation and management are crucial. Initial management involves assessing the patient's airway, with immediate attention given to patients with high levels of secretions as they are at high risk for aspiration.

Endoscopic instruments are available to aid in foreign body removal. Flexible endoscopes are preferred over rigid endoscopies due to the higher perforation rates associated with the latter [27-28]. An overtube can be advantageous, as it protects the airway while allowing for better endoscope passage [29].

**Figure 8:**
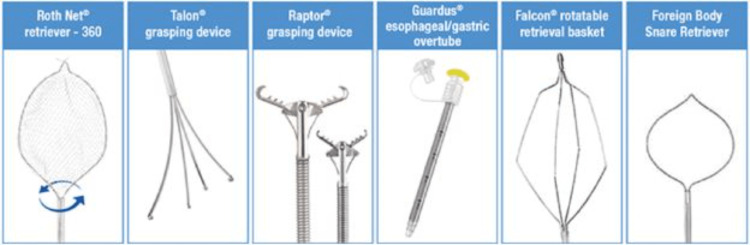
Reference STERIS (Dublin, Ireland)

Various tools can remove foreign bodies, including magnetic probes, grasping forceps, retrieval snare nets, transparent cap-fitting devices, and polypectomy snares. Retrieval nets, such as the Roth Net retriever, can encase objects entirely from the stomach or esophagus and protect the airway during removal. Grasping devices, like the rat tooth/alligator grasping forceps, Talon and Raptor grasping devices, and the Falcon retrieval basket (STERIS, Dublin, Ireland), can assist in removing foreign objects from both the upper and lower digestive tract (Figure 8) [30]. Balloon catheters, which have a balloon on the end that can be inflated, can also aid in foreign body retrieval. Finally, snares, which have a loop shape that can grab and remove objects from the digestive tract, have become widely employed to retrieve sharp or elongated objects [30-31].

Postoperative care

Endoscopic foreign body removal is a medical procedure that utilizes an endoscope to extract foreign objects ingested or lodged in the digestive tract. Although generally safe, there are possible complications and discomfort associated with the procedure [32-34,25].

Post-procedure care may involve various measures, such as monitoring the patient for complications and advising them to refrain from driving or operating heavy machinery for 24 hours. Additionally, the doctor may prescribe medications to alleviate any pain or discomfort the patient may experience. The patient may also be advised to follow a specific diet to aid in the healing of the digestive tract and drink plenty of fluids to prevent dehydration. Lastly, scheduling a follow-up appointment with the doctor is crucial to ensure no further complications [32-34,25].

Complications and management

Foreign body ingestion is a frequent emergency room diagnosis with high-risk populations, including patients with psychiatric illness, drug or alcohol abuse, elderly patients, and children [18]. While many cases of foreign body ingestion will pass spontaneously, approximately 10-20% of patients will require clinical intervention, with around 1% requiring surgical intervention [35,36]. Diagnosis is usually based on history and radiologic evidence [37].

The mainstay of treatment for foreign body ingestion is endoscopic removal of the foreign body, with longer durations between ingestion and endoscopy correlating with increased complications [38]. The complication rate after endoscopic foreign body removal can be as high as 5%, with factors such as the size and characteristics of the foreign body increasing the risk [38]. The most common complications include perforation, bleeding, infection, and airway compromise [36].

Perforations are a frequent complication, especially with sharp objects such as fish bones localized in the esophagus [18,37]. Symptoms of perforation after endoscopic intervention can present with erythema, tenderness, or crepitus [18]. Sharp objects that have passed into the stomach should be monitored with daily radiographs [18]. Surgical intervention may be necessary if perforation is suspected or progression has not occurred in 72 hours [18].

Bleeding is not uncommon during endoscopic procedures and is a minor complication [39], but laceration and associated bleeding are more common after removing sharp foreign bodies [37]. Infection is a minor and rare complication managed conservatively with empiric antibiotics [37]. The complication rate increases if intervention is delayed by more than 24 hours [36]. Therefore, early identification and endoscopic intervention in patients with foreign body impaction are crucial to improving clinical outcomes [37].

## Conclusions

In conclusion, foreign body ingestion is common and can lead to serious medical emergencies. This case study highlights the successful management of a patient who presented to the hospital with a foreign body lodged in his esophagus. The medical team's prompt and decisive action, including emergent endoscopic removal of the foreign body and careful postoperative management, ensured the patient's successful recovery without any postoperative complications. The importance of a multidisciplinary approach to managing such cases cannot be overstated, with close collaboration between medical, surgical, and psychiatric teams vital to achieving optimal outcomes. Additionally, this case emphasizes the need for patient education on the risks associated with foreign body ingestion, particularly for those with underlying psychiatric conditions. Finally, it is essential to recognize the importance of preventive measures and to implement appropriate interventions to minimize the risk of foreign body ingestion in vulnerable populations. Overall, this case study underscores the importance of prompt and coordinated action in managing foreign body ingestion and the critical role of healthcare providers in ensuring positive outcomes.
